# Combining CD4 count, CD8 count and CD4/CD8 ratio to predict risk of mortality among HIV-positive adults after therapy: a group-based multi-trajectory analysis

**DOI:** 10.3389/fimmu.2023.1269650

**Published:** 2023-12-06

**Authors:** Jing Ma, Guoyong Wang, Xiaoyan Zhu, Ling Li, Lin Wang, Lianzheng Hao, Lijie Gao, Wei Ma, Na Zhang

**Affiliations:** ^1^ Department of Epidemiology, School of Public Health, Cheeloo College of Medicine, Shandong University, Jinan, Shandong, China; ^2^ Institute of Preventive Medicine, Shandong University, Jinan, Shandong, China; ^3^ Institute for Acquired Immunodeficiency Syndrome (AIDS) Control and Prevention, Shandong Center for Disease Control and Prevention, Jinan, Shandong, China

**Keywords:** CD4 count, CD8 count, CD4/CD8 ratio, HIV, antiretroviral therapy, mortality

## Abstract

**Introduction:**

Previous studies have indicated different immunological recovery trajectories based on CD4 count or CD4/CD8 ratio. However, these immune indicators are interconnected, and relying solely on one indicator may lead to inaccurate estimates. Therefore, it is essential to develop a comprehensive trajectory model that integrates CD4 count, CD8 count and CD4/CD8 ratio.

**Methods:**

We utilized a group-based multi-trajectory model to characterize the latent cluster of recovery based on measurements of CD4 count, CD8 count and CD4/CD8 ratio over a period of up to 96 months following ART initiation. Subsequently, we investigated the characteristics associated with trajectory groups, especially sex and age. Cox model and Kaplan-Meier survival curve were employed to assess differences in all-cause, AIDS-related and non-AIDS related mortality between trajectory groups.

**Results:**

A total of 14,718 eligible individuals were followed for a median of 55 months. Longitudinal model identified four subgroups: group 1 (32.5%, low CD4 and CD4/CD8 inversion), group 2 (25.9%, high CD8 and CD4/CD8 inversion), group 3 (27.2%, slow recovery of CD4 and CD4/CD8 inversion) and group 4 (14.4%, rapid increase of CD4 and normal CD4/CD8). Immune recovery was slower in male than in female, and in elders than in youngers. Compared to group 2, group 1 (adjusted hazard ratio [aHR]=3.28; 95% CI 2.33-4.60) and group 3 (aHR=1.56; 95% CI 1.09-2.24) had increased risk of all-cause mortality after adjusting for other factors. Besides, group 1 (aHR=2.17) and group 3 (aHR=1.58) had higher risk of non-AIDS related mortality, and group 1 (aHR=5.92) had significantly increased risk of AIDS related mortality.

**Conclusion:**

Longitudinal trajectory analysis of multiple immune indicators can be employed to guide targeted interventions among vulnerable populations in clinical practice.

## 1 Introduction

Antiretroviral therapy (ART) substantially suppresses HIV viral replication, promotes immune reconstitution, and reduces the incidence of opportunistic events and mortality (1, 2). However, different patterns of immunologic recovery over time have been identified among HIV-infected patients receiving ART, and poor immune reconstitution is associated with prognosis such as death (3). A prior study reported that about 10%-40% of HIV/AIDS have not achieved normal immune system function, despite the success of viral load suppression (4). Therefore, identifying the trajectories of immune recovery and characteristics of high-risk groups among HIV-infected patients after receiving ART has significant programmatic implications.

CD4+ T-lymphocyte (CD4) counts have been found to be one of the strongest predictors of immunological recovery and disease progression after initiating ART among people living with HIV (PLWH) (5, 6); the majority of AIDS-related deaths occur in patients with CD4 count less than 350 cells/µL (7). Previous studies have found that CD4 cell counts increases rapidly over the first 3-6 months of ART (8, 9), but there is no clear consensus regarding long-term trends and maximum attainable CD4 levels. Some studies have reported that CD4 cell count stabilization occurs after 2-3 years or 4-5 years of ART, even after 5 years of treatment (10, 11).

In addition to CD4 counts, the CD8+ T-lymphocyte (CD8) counts and the CD4/CD8 ratio have been increasingly recognized as important makers of clinical progression and immunological reconstitution in recent years (12, 13). Furthermore, the CD4/CD8 ratio is an indicator of age-related cumulative inflammation and immunological alterations (14). Many studies have identified independent correlations between the CD8 cell counts or the CD4/CD8 ratio and the risk of mortality and non-AIDS events (12, 15, 16). Although CD8 counts remain elevated in chronic infections like HIV, they also rise in response to acute infections. Additionally, CD8 counts react to ART less quickly than CD4 counts.

Although several studies have assessed the potential trajectories of CD4 count or CD4/CD8 ratio for recovery (17, 18), relying solely on one indicator might lead to an overestimation of treatment effectiveness and immunological reconstitution. Additionally, these indicators are interconnected, making it difficult to measure the impact of a sole indicator. Previous research has demonstrated that high CD8 counts were associated with a poor increase in CD4 T-cells during ART (19). It is challenging to precisely determine the effect of CD8 cell counts independently of CD4 counts since high CD8 counts may be a homeostatic reaction to low CD4 counts (20). Consequently, it is crucial to develop a comprehensive trajectory model that incorporates prognostic markers such as CD4 count, CD8 count, and CD4/CD8 ratio.

Conventional statistical practice generally falls far short of taking full advantage of the available information in multivariable longitudinal data on illness progression. Fortunately, group-based multi-trajectory analysis (GBMTA) offers a promising method that has been underutilized. GBMTA identifies latent groups of individuals following similar trajectories that involve multiple interrelated indicators. It also transparently represents the interrelationship among clinically relevant indicators (21).

In this study, we employed group-based multi-trajectory models to characterize the timing and extent of CD4 count, CD8 count and CD4/CD8 ratio recovery over a period of up to 96 months among HIV positive individuals in Shandong Province, China. Subsequently, we estimated the association between distinctive recovery patterns and all-cause, AIDS related and non-AIDS related mortality.

## 2 Methods

### 2.1 Study design and participants

We conducted a retrospective cohort study in Shandong Province, China. Data were derived from the China’s National Free Antiretroviral Treatment Program (NFATP) database, a nationwide reporting system. In this analysis, we included HIV-infected adults who were aged 15 years or older at the initiation of ART, resided in Shandong Province, newly initiated ART between January 2004 and December 2020, and had at least three simultaneous measurements of CD4 cell count and CD8 cell count during the course of treatment. For all eligible patients, we obtained follow-up data until 31 December 2021, death or loss to follow-up.

### 2.2 Measurements

Measurements were obtained from the NFATP database. After each patient was diagnosed with HIV and gave consent to start ART, a standard assessment was conducted to collect baseline sociodemographic information and HIV/ART-related information. Detailed information on the NFATP has been published elsewhere (22). Patients in ART care were provided with free CD4 and CD8 testing, and the frequency of testing determined by national guideline for ART management among PLWH in China. Typically, CD4 testing was provided at least once per year. As a result, we collected data on CD4 cell counts, CD8 cell counts, and the dates of measurement between ART initiation and the end of follow-up period.

### 2.3 Categorization of cause of death

Deaths were classified on the basis of reported cause from the NFATP database and compared with the Coding of Death in HIV (CoDe) protocol (23). Deaths resulting from AIDS or immunodeficiency conditions were classified as “AIDS related mortality”. With respect to the CoDe protocol, AIDS related mortality included three categories: opportunistic diseases, cancers such as cervical cancer, and other causes related to HIV/AIDS. The detailed causes of HIV-related death among subjects were summarized in **Supplementary Table S1**. Individuals for whom information in the NFATP database was insufficient to determine the cause of death, or deaths with unknown cause, were categorized based on their last CD4 count and other AIDS-defining conditions prior to death. Specifically, deaths were classified as AIDS-related if patients had a low CD4 count (<100 cells/μL) within 90 days before death and a diagnosis consistent with AIDS and/or an AIDS-defining condition(s) close to death (details in **Supplementary Figure S1**). All other deaths (excluding AIDS-related deaths) were assumed to be non-AIDS related.

### 2.4 Statistical analysis

#### 2.4.1 Group-based multi-trajectory model

First, we employed group-based multi-trajectory analysis to identify distinct patterns of immunological recovery based on CD4 count, CD8 count, and CD4/CD8 ratio within 96 months after initiating ART. Detailed information about the GBMTA method can be found elsewhere (21, 24). Briefly, GBMTA is a technical form of finite mixture modeling that identifies unique clusters of individual recovery patterns within a given sample. This approach determines the best-fit solution using observed Bayesian information criteria (BIC) value through a multinomial modeling strategy. Since the number of groups and the order of the trajectory polynomials (i.e., linear, quadratic, cubic) are not known *a priori* (but must be pre-specified during model estimation), we systematically explored a series of model specifications, first by varying the number of groups (from two to five clusters) and then by adjusting the order of the trajectory polynomials. The optimum GBMTA model was determined based on the BIC value, high average posterior probabilities of group membership (>0.7), adequate patient representation (>5% in proportion) in each group, and clinical knowledge of the CD4 count and CD8 count (detailed in **Supplementary Tables S2**, **S3**) (25, 26).

#### 2.4.2 Baseline factors associated with trajectory group membership

We characterized patients in each trajectory group by summarizing baseline patient sociodemographic, clinical, and ART related variables. According to recommended free ART regimens for HIV-infected adults in China, the baseline regimens were classified into three categories: first-line regimen (3TC+AZT/TDF+EFV/NVP), second-line regimen (3TC+AZT/TDF+LPV/r) and others. The characteristics of individuals at enrollment were described using medians and interquartile ranges for continuous variables and frequency and proportions for categorical data. We utilized the chi-squared test for categorical variables to compare characteristics between the trajectory subgroups defined by the GBMTA model (**Table 1**). To explore the characteristics of age and sex among trajectory groups, we presented the distribution of trajectory groups within each age group, stratified by sex.

**Table 1:** Baseline characteristics by trajectory groups.

Characteristic	No. (%)					P-value
	Overall	Trajectory Group				
		Group 1	Group 2	Group 3	Group 4	
No. (%)	14718	4790(32.6)	3812(25.9)	4008(27.2)	2108(14.3)	
Gender						<0.001
Male	13236(89.9)	4357(91.0)	3521(92.4)	3522(87.9)	1836(87.1)	
Female	1482(10.1)	433(9.0)	291(7.6)	486(12.1)	272(12.9)	
Age at ART initiation(years), median (IQR)	33(27,43)	36(29,45)	30(26,38)	33(27,44)	34(28,44)	
Age group(years)						<0.001
15-29	5586(38.0)	1386(29.0)	1906(50.0)	1533(38.2)	761(36.1)	
30-39	4472(30.4)	1558(32.5)	1138(29.8)	1158(28.9)	618(29.3)	
40-49	2786(18.9)	1103(23.0)	483(12.7)	752(18.8)	448(21.3)	
≥50	1874(12.7)	743(15.5)	285(7.5)	565(14.1)	281(13.3)	
Marriage status ^a^						<0.001
Unmarried	6624(45.0)	1825(38.1)	2151(56.4)	1753(43.7)	895(42.5)	
Married	5577(37.9)	2031(42.4)	1132(29.7)	1570(39.2)	844(40.0)	
Divorced/widowed	2515(17.1)	934(19.5)	528(13.9)	684(17.1)	369(17.5)	
Education level ^b^						<0.001
Senior school and below	5896(40.1)	2138(44.7)	1197(31.4)	1674(41.8)	887(42.1)	
Middle school	3934(26.7)	1283(26.8)	1069(28.1)	1045(26.1)	537(25.5)	
High school and above	4882(33.2)	1366(28.5)	1544(40.5)	1288(32.1)	684(32.4)	
Transmission route						<0.001
Heterosexual	4375(29.7)	1571(32.8)	901(23.7)	1259(31.4)	644(30.5)	
Homosexual	9765(66.4)	2966(61.9)	2803(73.5)	2592(64.7)	1404(66.6)	
Others	578(3.9)	253(5.3)	108(2.8)	157(3.9)	60(2.9)	
WHO stage						
1/2	13257(90.1)	3736(78.0)	3687(96.7)	3780(94.3)	2054(97.4)	
3/4	1461(9.9)	1054(22.0)	125(3.3)	228(5.7)	54(2.6)	
Calendar period of ART initiation						<0.001
≤2015	4807(32.7)	1832(38.2)	1274(33.4)	1241(31.0)	460(21.8)	
2016-2018	7107(48.3)	2035(42.5)	1950(51.2)	1946(48.5)	1176(55.8)	
2019-2021	2804(19.0)	923(19.3)	588(15.4)	821(20.5)	472(22.4)	
Nadir CD4 count (cells/µL), median (IQR)	352(205,503)	154(48,287)	458(354,590)	338(251,445)	521(403,678)	
Nadir CD4 count category, cells/µL						<0.001
≤200	3594(24.4)	2850(59.5)	156(4.1)	544(13.6)	44(2.1)	
201-350	3725(25.3)	1070(22.4)	757(19.9)	1599(39.9)	299(14.2)	
351-500	3673(25.0)	524(10.9)	1319(34.6)	1205(30.0)	625(29.6)	
>500	3726(25.3)	346(7.2)	1580(41.4)	660(16.5)	1140(54.1)	
ART regimen at baseline						<0.001
First-line(3TC+AZT/TDF+EFV/NVP)	13809(93.8)	4396(91.8)	3613(94.8)	3798(94.8)	2002(95.0)	
Second-line(3TC+AZT/TDF+LPV/r)	326(2.2)	94(2.0)	97(2.5)	73(1.8)	62(2.9)	
Others	583(4.0)	300(6.2)	102(2.7)	137(3.4)	44(2.1)	

*P* values were calculated using χ2 test.

a 2 missing value.

b 6 missing value.

IQR, interquartile range; WHO, World Health Organization; ART, antiretroviral therapy; AZT, zidovudine; 3TC, lamivudine; EFV, efavirenz; NVP, Nevirapine; TDF, tenofovir.

#### 2.4.3 Mortality by trajectory group membership

We aimed to evaluate differences in mortality based on trajectory group membership. Initially, we employed Cox proportional hazard models to estimate unadjusted, adjusted (for age group at ART initiation, sex, WHO clinical stage, calendar period of ART initiation, baseline regimen) and additionally adjusted (for baseline CD4 count) mortality hazard ratios for trajectory groups. Separate models were estimated for all-cause, AIDS related, and non-AIDS related mortality. Those who died from non-AIDS related cause were censored at the date of death in the analysis of AIDS related mortality (and vice versa). Subsequently, we used the Kaplan-Meier survival curve to obtain stratified estimates of the cumulative incidence of mortality by trajectory groups. Time 0 was the date of ART initiation, and patients were administratively censored at the time of transfer or the end of observation.

#### 2.4.4 Sensitivity analysis

Given the potential impact of treatment failure on immune reconstitution, we conducted a subgroup analysis focusing on patients with available viral load (VL) records post-treatment. ART failure was defined based on the most recent VL record after six months of ART. Patients with a last VL greater than 50 copies/ml were categorized as virological unsuppressed (indicating treatment failure), while those with a last VL less than 50 copies/ml were classified as virological suppressed. To ensure the robustness of our finding, we conducted both GBMTA and Cox analyses on mortality among virologically suppressed and unsuppressed patients. Additionally, we compared the consistency of trajectory assignments for virologically suppressed and unsuppressed patients with their trajectory assignments in the overall cohort.

Analyses were carried out with SAS version 9.4 and R version 4.2.2.

### 2.5 Ethical approval statement

Since this was a secondary analysis of routine laboratory data, no written informed consent was obtained from patients. This study was reviewed and approved by Shandong University Ethics Review Committee of Public Health (IRB No:20220806).

## 3 Results

### 3.1 Patient characteristics

A total of 14,718 patients were included in our analysis, with a median follow-up time on ART of 55 months (Interquartile Range [IQR]: 37.0-75.3). Throughout the follow-up period, 459 (3.1%) patients died in a median time of 40.4 months (IQR: 23.2-65.8). The median age of the study participants was 33 (IQR: 27-43) years, with the majority being male (89.9%). Approximately 45% of the patients were unmarried, and roughly two-thirds were infected through homosexual intercourse (66.4%). At enrollment, the majority of patients presented with WHO clinical stage 1 or 2 (90.1%), and the median CD4 cell count was 352 cells/µL (IQR: 205-503). The primary regimens were first-line 3TC+AZT/TDF+EFV/NVP (93.8%) and second-line 3TC+AZT/TDF+LVP/r (2.2%) (see **Table 1**).

### 3.2 Description of immunological recovery trajectory groups

**Figure 1:**
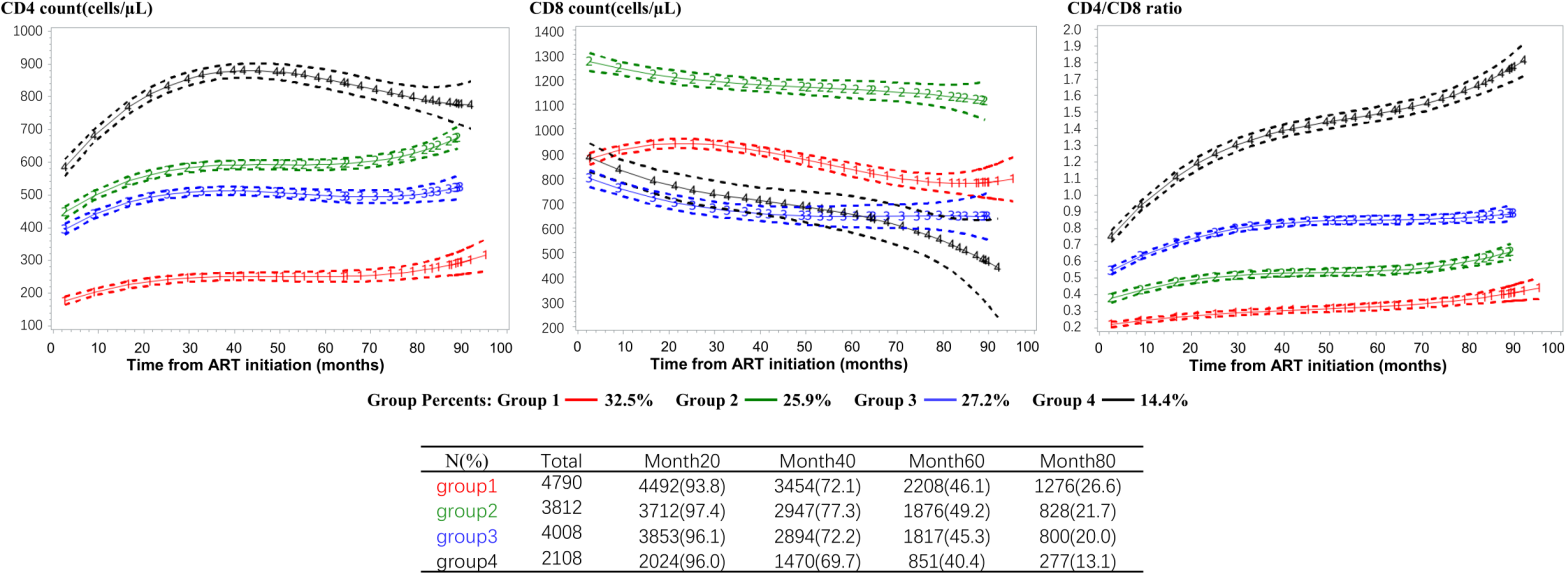
CD4 count, CD8 count and CD4/CD8 ratio trajectories defined by the group-based multi-trajectory analysis. CD4+ T-cell count (left column), CD8+ T-cell count (middle column), and CD4/CD8 T-cell ratio (right column) trajectories. The trajectories curves were estimated by group based multi-trajectory model, and the table below the graph shows the size of the risk set included in each group at 0, 20, 40, 60, 80 months after treatment. ART, antiretroviral therapy.

The optimal model identified four trajectory groups based on longitudinal patterns in changes of CD4 count, CD8 count and CD4/CD8 ratio within 96 months after ART initiation among PLWH (**Figure 1**). Group 1 (32.5% of participants) was characterized by a low CD4 count (<200 cells/µL) and high CD8 count with slow speed of recovery, and an inversion of CD4/CD8 ratio during follow up. Group 2 (25.9%) exhibited a medium-level CD4 count initially, which increased gradually, along with the highest CD8 count initially, which decreased slowly. Group 3 (27.2%) and group 2 presented an interesting contrast. The CD4 count trajectory in Group 3 was similar to that in Group 2, but the CD8 count was lower, resulting in a higher CD4/CD8 ratio compared to Group 2. Group 4 (14.4%) was characterized by a high baseline CD4 count (above 500 cells/µL), which increased rapidly during the first 30 months after ART initiation. Additionally, it had a low baseline CD8 count that decreased rapidly, and the CD4/CD8 ratio rose rapidly, reaching a normal level (1.4-2.0) after approximately 50 months. **Supplementary Tables S2**, **S3** provided diagnostic metrics for our final model, indicating a very good fit and excellent group separation based on well-established metrics.

### 3.3 Characteristics comparisons between trajectory groups

**Figure 2:**
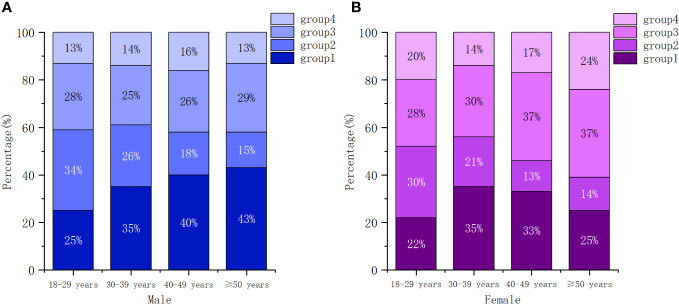
Conditional distributions of participants across trajectory groups by gender and age. **(A)** male, **(B)** female.

Our analysis revealed that baseline characteristics, including sex, age group, marital status, education level, transmission route, WHO clinical stage, calendar period of ART initiation, CD4 count category and ART regimen, were strongly correlated with trajectory group membership (see **Table 1**). Patients who were older, married, had lower education level, were in a later WHO clinical stage (3/4), initiated ART before 2016, and had a baseline CD4 count less than 200 cells/µL were more likely to belong to group 1. Conversely, younger patients (15-29 years), unmarried, with high education level, transmitted by homosexual, had a median level of CD4 count at enrollment (>350 cells/µL) were more likely to be classified in group 2. Participants who started ART between 2019 and 2021, had a baseline CD4 count exceeding 500 cells/µL, and received a second-line regimen (3TC+AZT/TDF+LPV/r) showed a tendency to be in group 4. Moreover, females were somewhat more likely to be associated with group 3 and group 4. Among older patients, older females were more likely to be in group 3, while older males were more likely to belong to group 1 (see **Figure 2**).

### 3.4 Mortality by trajectory group membership

**Figure 3:**
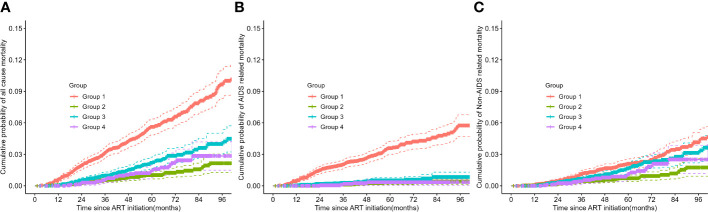
Kaplan-Meier survival plots of all-cause, AIDS related, and non-AIDS related mortality across trajectory subgroups. **(A)** all-cause mortality, **(B)** AIDS related mortality, and **(C)** non-AIDS related mortality. ART, antiretroviral therapy.

There were marked differences in mortality risks based on trajectory group membership (**Table 2**; **Figure 3**). As depicted in **Table 2**, univariate Cox regression analysis indicated that group 1 (hazard ratio [HR]=4.50; 95% CI 3.31-6.11) and group 3 (HR=1.82; 95% CI 1.28-2.59) were associated with higher all-cause mortality compared to group 2 (reference). Even after adjusting for age, sex, WHO clinical stage, calendar year of ART initiation, baseline regimen and baseline CD4, group 1 (adjusted hazard ratio [aHR]=3.28; 95% CI 2.33-4.60), and group 3 (aHR=1.56; 95% CI 1.09-2.24) exhibited significantly increased mortality risks compared to group 2. Concerning AIDS related mortality, the aHR for group 1 (vs group 2) was 5.92 (95% CI 3.26-10.77). Before adjusting for confounding variables, non-AIDS related mortality was higher in group 1 and group 3 (HR=2.57 [95% CI 1.75-3.76] and HR=1.99 [95% CI 1.32-3.00], respectively) compared to group 2. After full adjustment, these associations attenuated to 2.17 (95% CI 1.42-3.32) and 1.58 (95% CI 1.03-2.41), respectively.

**Table 2:** Mortality hazard ratios for all-cause, AIDS related, and non-AIDS related deaths across trajectory model.

Mortality	Group 1	Group 2	Group 3	Group 4	P value
All-cause mortality					
Unadjusted	4.50(3.31-6.11)	1	1.82(1.28-2.59)	1.41(0.90-2.21)	<.0001
Adjusted, no CD4 ^a^	3.60(2.64-4.92)	1	1.54(1.08-2.19)	1.24(0.79-1.94)	<.0001
Fully adjusted ^b^	3.28(2.33-4.60)	1	1.56(1.09-2.24)	1.24(0.79-1.95)	<.0001
AIDS related mortality					
Unadjusted	9.32(5.40-16.09)	1	1.41(0.71-2.78)	0.73(0.26-2.03)	<.0001
Adjusted, no CD4 ^a^	7.74(4.45-13.47)	1	1.31(0.66-2.60)	0.74(0.27-2.05)	<.0001
Fully adjusted ^b^	5.92(3.26-10.77)	1	1.36(0.68-2.73)	0.75(0.27-2.09)	<.0001
Non-AIDS related mortality					
Unadjusted	2.57(1.75-3.76)	1	1.99(1.32-3.00)	1.74(1.05-2.89)	<.0001
Adjusted, no CD4 ^a^	2.02(1.37-2.98)	1	1.55(1.02-2.34)	1.35(0.81-2.24)	0.003
Fully adjusted ^b^	2.17(1.42-3.32)	1	1.58(1.03-2.41)	1.32(0.79-2.19)	0.003

Data are presented as hazard ratio (95% confidence interval) unless otherwise indicated. Deaths: all-cause=459; AIDS-related=207; non-AIDS-related=252.

a The same as the fully adjusted analysis but without adjustment for baseline CD4.

b Fully adjusted model includes age group, sex, WHO clinical stage, calendar year of ART initiation, baseline regimen and baseline CD4.

Consistent with the results of the Cox analysis, all-cause mortality was significantly higher in group 1 and group 3 compared to the other two groups (refer to **Figure 3**). The primary cause of death for patients in group 1 was AIDS related diseases, whereas for the other three groups, it was non-AIDS related. The probability of non-AIDS related mortality slightly increased after the third year of treatment.

### 3.5 Sensitivity analysis

The sensitivity analysis demonstrated that the assignment to trajectories remained relatively robust even among patients with treatment failure, with over 86% of these patients’ trajectory subgroups being consistent. Further details are provided in the **Supplementary Material** (**Figure S4**). Besides, the trajectory graph using CD4 count alone (see **Supplementary Figure S5**) exhibited broad similarities with the CD4 trajectory graph combining three metrics. However, the trajectory graph of CD4 count alone provided limited information, with four different trajectories essentially showing a parallel upward trend, and the trajectory subgroups aligning with baseline CD4 levels. Conversely, the trajectory graph combining three metrics illustrated the changes in CD4 count and CD8 count, as well as the correlations behind different CD4/CD8 ratio recovery trajectories. Further details are presented in the **Supplementary Material**.

## 4 Discussion

We observed significant heterogeneity in the immunological recovery trajectory by integrating multiple indicators among new ART starters in Shandong Province during 2004-2021. This suggests the potential for targeted interventions based on patients’ trajectory rather than a one-size-fits-all strategy. Utilizing a group-based multi-trajectory model, we identified four distinct individual subgroups: group 1 with a low CD4 count, high CD8 count at baseline and slow recovery (32.5%); group 2 with a medium level of CD4, high level of CD8 and slow recovery (25.9%); group 3 with a medium level of CD4, low CD8 and a middle level of CD4/CD8 (27.2%); and group 4 with a high CD4, low CD8 and persistently high level of CD4/CD8 (14.4%). Within our cohort, only patients in group 4 reached a normal CD4/CD8 ratio (>1.4) during 96-months follow-up. All-cause mortality and non-AIDS related mortality in group 1 and group 3 were higher than in other groups, even after adjusting for confounding variables, and AIDS related deaths were significantly higher in group 1.

It is well established that the recovery of the CD4/CD8 ratio is driven by rises in CD4 count and declines in CD8 count following ART initiation. Notably, there were two significantly different trajectories in CD8 cell counts among participants with similar baseline CD4 count (group 2 and group 4), leading to large differences in CD4/CD8 ratio trajectories between the two groups. Consistent with previous studies (17, 27), we found that the CD4 count at the start of ART was the strongest predictor of CD4 count recovery. The CD4 count in four trajectory groups all increased during the first several years of treatment, with faster gains among patients with a higher baseline CD4 count. Subsequently, CD4 cell recovery entered a plateau period with much slower and insignificant gains around three years post ART, except for group 1 with a baseline CD4 count of less than 200 cells/µL, which maintained moderate growth, and the other three groups returned to slow increases after the 7th year. Therefore, the optimal choice of the initial ART regimen is crucial for maximizing early CD4 gains. In contrast, the changes in CD8 cell count exhibited different trends. For group 1 with a higher baseline CD8 count (about 950 cells/µL), the CD8 count continued to increase during the initial two years of treatment and then began to decline.

We observed that baseline characteristics were associated with trajectory group memberships. In general, immune recovery is slower in men than in women, and in older patients than in younger patients (17, 28). Specifically, middle-aged and older individuals (>40 years) of male gender were more likely to belong to group 1, while female aged >40 years were more likely to belong to group 3. Compared with group 2, participants who were older, married, with lower level of education, and transmitted by heterosexual contact were more likely to belong to group 1 and group 3. Additionally, patients who were at a later WHO clinical stage at baseline were more likely to be in group 1. Furthermore, females and patients with a high baseline CD4 count were more likely to belong to group 4. Consistent with a previous study (29), patients who received regimens containing protease inhibitors (such as LPV/r) exhibited better immune reconstitution.

Undoubtedly, the mortality risk was highest in group 1 with a low CD4 count and high CD8 count profile. However, we found that all-cause mortality and non-AIDS related mortality were higher in group 3 (vs. group 2) even after adjusting for other prognostic factors at baseline. Individuals in group 3 may be overlooked in clinical practice, as this population had a moderate CD4 level and a normal CD8 level at baseline, suggesting that medical staff should pay attention to patients with this profile during follow-up. Furthermore, this also suggests the need to be cautious about the definition of immune non-responders (INRs). INRs have higher risk of opportunistic infections and mortality (3), but there are currently no universal criteria for INRs. In some studies, INRs were defined as either a CD4 cell count failing to reach a specified threshold (eg., CD4 <350 cells/µL) or a specified percentage of increase (4). According to the Department of Health and Human Services, INRs were defined as PLWH with a CD4 cell count still less than 350 or 500 cells/µL after 4-7 years of ART (4). However, the findings from our study, particularly group 3, indicate that the proportion of INRs might have been underestimated by this definition. It is preferable to define INRs using a higher CD4 count threshold (500 cells/µL) in the early years of ART or based on the absolute value of gain according to our findings.

While the expansion of CD8 cells is a classical hallmark of immunosenescence, a previous study has found that participants with increased CD8 cell counts were protected against infectious non-AIDS events (30). This performance is consistent with the characteristics of patients in group 2 in our study. Specifically, those who were younger male (aged 15-29 years), transmitted by homosexual intercourse, initiated ART before 2016, and received first-line regimen (3TC+AZT/TDF+EFV/NVP) were more likely to be in the high CD8 cell count group (group 2).

Uncovering trajectory groups defined by longitudinal measurements of CD4 count, CD8 count and CD4/CD8 ratio provides a richer understanding of PLWH immunological recovery and diseases progression as compared to traditional analysis and can help to identify distinctive recovery patterns. In comparison to traditional methods or trajectory analysis of a sole indicator, the trajectory groups identified by the GBMTA showed a stronger correlation with mortality (7, 31).

Our study also had several limitations. Firstly, the World Health Organization (WHO) updated treatment guidelines in 2019, recommending new drugs may accelerate immune reconstitution and improving prognosis. Therefore, the extrapolation of the findings of this study may be restricted. However, since most low- and middle-income countries are currently unable to switch medications, these findings are applicable and could provide recommendations for lower-middle income countries. Secondly, there have been numerous advances in HIV care during these years, such as universal testing and treatment, as well as variation in ART regimen, which may alter the distribution between trajectory groups. However, it is unlikely that they would dramatically change the general immune patterns underlying each trajectory. Thirdly, our analysis of factors associated with assignment to trajectories was limited to the information available in the data from NFATP. Other important factors such as ART resistance, duration of infection and clinical infections information may affect the assignment to trajectories. To some extent, nadir CD4 counts at enrollment reflect the duration of infection, and we included baseline CD4 counts in this study. Further investigation is needed to determine. Lastly, our results can’t be used directly for risk prediction for new patients receiving ART, as we currently don’t provide an algorithm that assigns new patients to one of the four trajectory groups.

In summary, our study identified four distinct trajectory groups based on longitudinal changes in CD4 count, CD8 count and CD4/CD8 ratio within 96 months after ART initiation among PLWH. More than half of individuals did not achieve a CD4/CD8 ratio ≥1.0 during follow-up. The immunological recovery was slower in men than in women, and in older patients than in younger patients. The recovery trajectories may be one of critical predictors of mortality risk. Thus, longitudinal trajectory analysis of multiple immune indicators can be used to guide targeted interventions among vulnerable populations in clinical practice.

## Data availability statement

The original contributions presented in the study are included in the article/**Supplementary Material**; further inquiries can be directed to the corresponding author/s.

## Ethics statement

The studies involving humans were approved by Shandong University Ethics Review Committee of Public Health. The studies were conducted in accordance with the local legislation and institutional requirements. The human samples used in this study were acquired from a by- product of routine care or industry. Written informed consent for participation was not required from the participants or the participants’ legal guardians/next of kin in accordance with the national legislation and institutional requirements.

## Author contributions

JM: Conceptualization, Data curation, Formal analysis, Methodology, Software, Visualization, Writing – original draft, Writing – review & editing. GW: Conceptualization, Project administration, Resources, Supervision, Validation, Writing – review & editing. XZ: Supervision, Validation, Writing – review & editing, Data curation, Investigation. LL: Data curation, Investigation, Supervision, Validation, Writing – review & editing. LW: Investigation, Validation, Writing – review & editing, Visualization. LH: Investigation, Writing – review & editing, Data curation, Supervision. LG: Supervision, Writing – review & editing, Methodology, Validation. WM: Conceptualization, Funding acquisition, Methodology, Project administration, Resources, Supervision, Validation, Writing – review & editing. NZ: Conceptualization, Data curation, Funding acquisition, Investigation, Project administration, Resources, Supervision, Validation, Writing – review & editing.
