# Supplementary material for: Combining CD4 count, CD8 count and CD4/CD8 ratio to predict risk of mortality among HIV-positive adults after therapy: a group-based multi-trajectory analysis
Source: Front Immunol. 2023 Dec 6;14:1269650. doi: 10.3389/fimmu.2023.1269650 (PMC10731246; doi:10.3389/fimmu.2023.1269650)
Supplement: Supplementary file 1 [file DataSheet_1.docx]

***Supplementary Material***

1. **Categorization of cause of death**

We used an algorithm to classify cause of deaths (see Figure S1). Firstly, for primary cause of death reported as an AIDS-defining illness in database, deaths were labeled “AIDS related” (n=115, the specific causes of death as shown in Table S1). Secondly, patients with CD4 count of <100 cells/µL within three months of death were identified as AIDS-related deaths (n=38). Thirdly, patients were classified as AIDS-related deaths if they fulfilled two or more of the following conditions at the same time: CD4<100 cells/µL within one year of death, AIDS within one year of death, less than three months between HIV diagnosis and death, age of <= 60 years at death, and the last seen for care more than 12 months prior to death (n=54). All other deaths were assumed to be non-AIDS related. Based on the above rules, there were 207 patients classified as AIDS-related deaths and the other deaths were classified as non-AIDS-related (n=252).

Consider coding death as AIDS related if:

Ⅱ. If primary cause of death was insufficient to determine the cause, with

1. CD4 count of <100 cells/µL within 90 days of death (n=38)
2. Two or more of the following (n=54):
3. CD4 <100 cells/µL within one year of death
4. AIDS within one year of death
5. Less than three months between HIV diagnosis and death
6. Age of ≤60 at death
7. Last seen for care >12 months prior to death

Ⅰ. Primary cause of death reported as an AIDS-defining illness (n=115)

**Figure S1. Algorithm to classify cause of deaths.**

**Table S1. Detail causes of AIDS related deaths in database.**

| **Detailed causes of AIDS-related deaths** | **Number** | **Percent (%)** |
| --- | --- | --- |
| Mycobacterium tuberculosis infection (intrapulmonary) | 3 | 2.6 |
| Cytomegalovirus infection (other than liver, spleen, lymph nodes) | 1 | 0.9 |
| Candida infections (esophagus or lung, trachea, bronchus) | 4 | 3.5 |
| Recurring bacterial pneumonia | 8 | 7.0 |
| Pneumocystis carinii pneumonia | 13 | 11.3 |
| Cryptococcosis (cryptococcal meningitis, extrapulmonary cryptococcosis) | 4 | 3.5 |
| Acute AIDS Infection Syndrome | 6 | 5.2 |
| Wasting syndrome | 8 | 7.0 |
| HIV encephalopathy (AIDS-related encephalopathy or dementia syndrome) | 7 | 6.1 |
| Invasive cervical cancer | 1 | 0.9 |
| Burkitt's lymphoma | 1 | 0.9 |
| Hodgkin's lymphoma | 1 | 0.9 |
| Other non-Hodgkin's lymphoma (brain or B-cell) | 3 | 2.6 |
| Other AIDS-related tumors | 12 | 10.4 |
| Multiple malignant neoplasms (metastatic tumors, lymphoma undiagnosable) | 9 | 7.8 |
| Other AIDS-related specified diseases and syndromes | 7 | 6.1 |
| Missing | 27 | 23.5 |
| Total | 115 | 100 |

**2. Steps of** **the group-based multi-trajectory model (GBMTM) and assessment of the model**

The group based multi-trajectory model (GBMTM) employs maximum likelihood estimation to determine both the trajectory shape for each group (modeled as a complex function of the biomarkers and time) and the estimated probabilities of each individual belonging to each trajectory group based on their observed data. Subsequently, each individual is assigned to a specific group based on the highest probability of group membership. The number of trajectory groups and the order of the polynomials of the time function are not known a priori and are determined through systematic model search, following the guidelines outlined in Nagin et al [1-2].

In the initial step of GBMTM, trajectory models were separately estimated with varying numbers of groups (ranging from two to five clusters) for biomarkers to identify distinct trajectories within the overall population. The subsequent step involved varying the order of the trajectory polynomials (linear, quadratic, cubic) of time, without including interaction terms.

We assessed the final GBMTM in terms of adequacy and fit of the trajectories using these criteria: (1) lower Bayesian Information Criterion (BIC), (2) high average posterior probabilities of group membership (>0.7), (3) substantial proportion of patients (>5%) classified in each group, and (4) narrow confidence bands for each group [1, 3-5]. Additionally, alongside the statistical rationale employed in GBMTM, clinical context was taken into account when grouping trajectories in our analyses. For instance, in individuals without HIV infection, a typical CD4 range is between 500 and 1000 cells/μL [6]. Likewise, people living with HIV (PLWH) with CD4 counts greater than 500 cells/μL are generally considered to have experienced good immune recovery. Consequently, in line with prior literature [7], our analysis grouped all trajectory CD4 counts at and above 500 cells/μL into a single trajectory.

Based on the BIC value and prior knowledge, we selected the optimal model with four trajectory groups (Table S2). Subsequently, we assessed the model's performance using the following criteria: (1) close agreement between the model’s estimated group size and the actual percentage of patients classified into each group, (2) high average posterior probabilities of group membership (>0.7), and (3) substantial proportion of patients (>5%) classified in each group (Table S3). These metrics indicated that it had very good fit and excellent group separation.

**Table S2. The Bayesian Information Criterion of model varying the number of groups (from two to five clusters).**

| Number of trajectory group | Order of the trajectory polynomials | Bayesian Information Criterion | Akaike information criterion |
| --- | --- | --- | --- |
| 2 | (3,3) | -1703901 | -1703794 |
| 3 | (3,3,3) | -1680213 | -1680057 |
| 4 | (3,3,3,3) | -1663314 | -1663109 |
| 5 | (3,3,3,3,3) | -1672078 | -1671818 |

**Table S3. Performance of the optimum model for the trajectories.**

| Trajectory group | Estimated group size by model, % | No. of patients classified into group | % of patients classified into group | Average posterior probabilities of group membership |
| --- | --- | --- | --- | --- |
| 1 | 32.5 | 4790 | 32.5 | 0.966 |
| 2 | 25.9 | 3812 | 25.9 | 0.939 |
| 3 | 27.2 | 4008 | 27.2 | 0.930 |
| 4 | 14.4 | 2108 | 14.4 | 0.966 |
| Total | 100.0 | 14718 | 100.0 |  |

[1] Nagin DS, Jones BL, Passos VL, Tremblay RE. Group-based multi-trajectory modeling. Stat Methods Med Res 2018; 27:2015-2023.

[2] Nagin D. Group-based modeling of development. Cambridge: Harvard University Press; 2005.

[3] Mody A, Eshun-Wilson I, Sikombe K, Schwatz SR, Beres LK, Simbeza S, et al. Longitudinal engagement trajectories and risk of death among new ART starters in Zambia: A group-based multi-trajectory analysis. Plos Med 2019;16: e1002959.

[4] Nagin DS, Odgers CL. Group-based trajectory modeling in clinical research. Annu Rev Clin Psvchol 2010; 6:1091384.

[5] Kliin SL, Weljenberg MP, Lemmens P, van den Brandt PA, Lima PV. Introducing the fit-criteria assessment plot a visualisation tool to assist class enumeration in group-based trajectory modelling. Stat Methods Med Res 2017; 26:2424-2436.

[6] Moir S, Chun TW, Fauci AS. Pathogenic mechanisms of HIV disease. Annu Rev Pathol 2010; 6:223–248.

[7] Lok JJ Bosch RJ Benson CA, et al. Long-term increase in CD4+ T-cell counts during combination antiretroviral therapy for HIV-1 infection. AIDS 2010; 24:1867-1876.

**3. Sensitivity analysis**

**3.1 Impact of failure of treatment on trajectory groups**

Among the entire eligible population (N=14718), 14518 cases (98.6%) had at least one viral load result after 180 days of treatment and were included in the subset analysis. Failure of antiretroviral therapy (ART) was determined based on the last viral load recorded after six months of ART, with patients having a last viral load greater than 50 copies/ml defined as virologically unsuppressed (13197), and those with a last viral load less than 50 copies/ml defined as virologically suppressed (1321).

We observed that the trajectory graphs were similar between the aforementioned two groups (Figure S2 represented the trajectory group among patients with virological suppression, and Figure S3 represented the trajectory group among patients with virological unsuppressed). The proportion of the worst immune recovery group was higher in patients who failed to achieve virological suppression compared to those who were virologically suppressed (42.6% vs. 31.7%). Sensitivity analysis demonstrated that the assignment of immune reconstruction trajectories remained relatively robust even among patients with treatment failure, with over 86% of those patients’ trajectory subgroups showing consistency (Figure S4).

In comparison with group 1 among virologically suppressed patients, group 1 among treatment-failed patients had a higher likelihood of AIDS-related deaths and a similar likelihood of non-AIDS-related deaths (Table S4).

**Figure S2. CD4 count, CD8 count and CD4/CD8 ratio trajectories among patients with viral load suppressed(n=13197).**


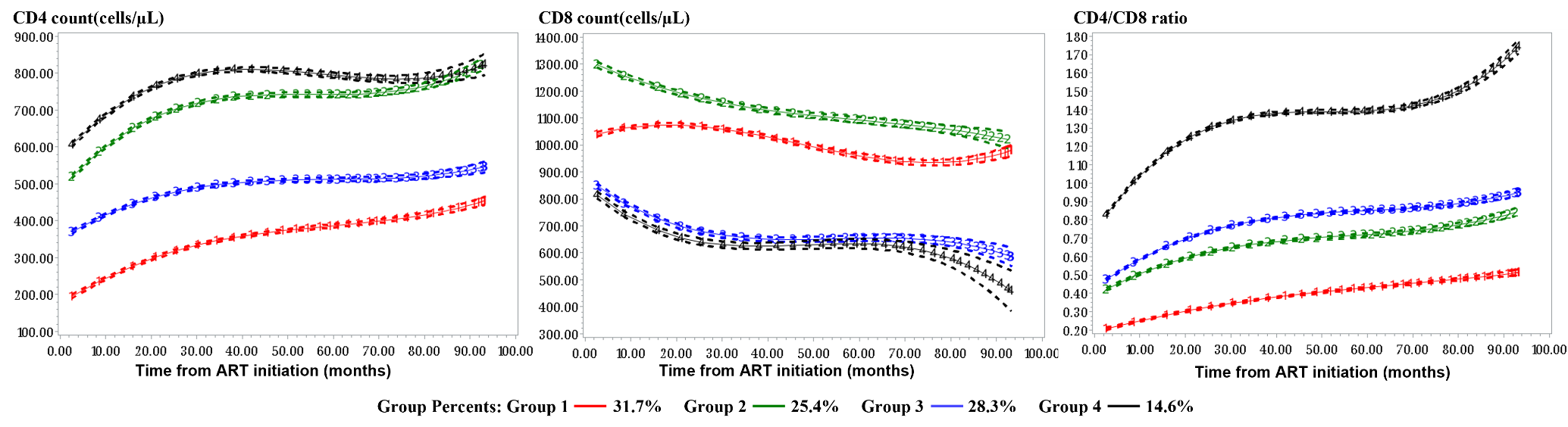


**Figure S3. CD4 count, CD8 count and CD4/CD8 ratio trajectories among patients with viral load unsuppressed(n=1321).**


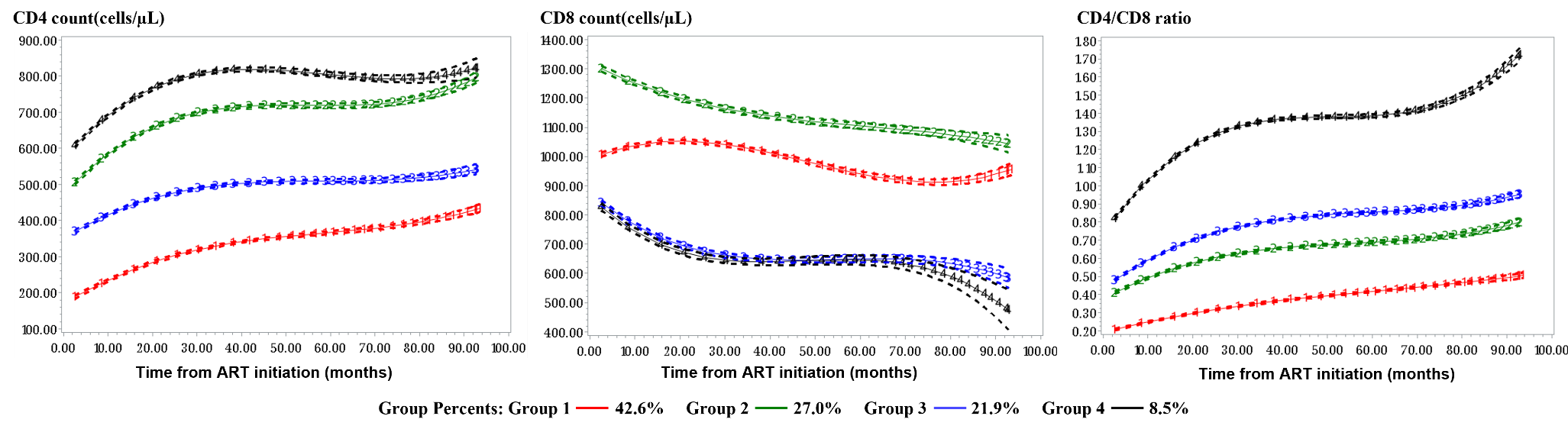


**Figure S4. Distribution of the original all-patient trajectory group in the trajectory subgroup for treatment-failure patients.**

**Table S4. Mortality hazard ratios for all-cause, AIDS related, and non-AIDS related deaths among patients with viral load suppressed and unsuppressed.**

|  | **Patients with viral load suppressed** | | | | | **Patients with viral load unsuppressed** | | | |
| --- | --- | --- | --- | --- | --- | --- | --- | --- | --- |
| Mortality | Group 1 | Group 2 | Group 3 | Group 4 | Group 1 | | Group 2 | Group 3 | Group 4 |
| **All-cause mortality** |  |  |  |  |  | |  |  |  |
| Unadjusted | 2.52(1.73-3.69)*** | 1 | 1.99(1.33-2.98)*** | 1.66(1.01-2.75)* | 6.47(3.28-12.72)*** | | 1 | 1.44(0.62-3.37) | 1.34(0.41-4.35) |
| Adjusted, no CD4 | 1.91(1.30-2.82)** | 1 | 1.57(1.04-2.36)* | 1.32(0.80-2.19) | 6.32(3.18-12.57)*** | | 1 | 1.40(0.59-3.30) | 1.41(0.43-4.64) |
| Fully adjusted | 1.99(1.29-3.05)** | 1 | 1.62(1.07-2.46)* | 1.30(0.78-2.15) | 4.94(2.40-10.14)*** | | 1 | 1.31(0.55-3.12) | 1.32(0.40-4.38) |
| **AIDS related mortality** |  |  |  |  |  | |  |  |  |
| Unadjusted | 3.92(1.91-8.05)*** | 1 | 1.31(0.55-3.11) | 0.93(0.28-3.01) | 14.20(4.49-44.89)*** | | 1 | 1.69(0.40-7.06) | 1.04(0.11-9.99) |
| Adjusted, no CD4 | 2.86(1.37-6.00)** | 1 | 1.10(0.46-2.61) | 0.76(0.23-2.50) | 14.13(4.44-44.96)*** | | 1 | 1.77(0.42-7.47) | 1.25(0.13-12.11) |
| Fully adjusted | 2.40(1.04-5.50)* | 1 | 1.11(0.46-2.71) | 0.78(0.24-2.57) | 9.71(2.95-31.96)*** | | 1 | 1.63(0.38-6.93) | 1.12(0.12-11.00) |
| **Non-AIDS related mortality** |  |  |  |  |  | |  |  |  |
| Unadjusted | 2.06(1.31-3.23)** | 1 | 2.22(1.40-3.52)*** | 1.93(1.10-3.39)* | 2.64(1.11-6.27)* | | 1 | 1.29(0.45-3.73) | 1.41(0.35-5.67) |
| Adjusted, no CD4 | 1.59(1.01-2.52)* | 1 | 1.72(1.08-2.73)* | 1.52(0.86-2.68) | 2.37(0.97-5.81) | | 1 | 0.98(0.33-2.92) | 1.13(0.27-4.71) |
| Fully adjusted | 1.81(1.09-3.00)* | 1 | 1.79(1.12-2.89)* | 1.47(0.83-2.59) | 2.38(0.91-6.20) | | 1 | 1.00(0.33-3.03) | 1.11(0.27-4.68) |

**3.2 Trajectory of combining three metrics vs. using CD4 count alone**

The trajectory results showed that the trajectory graph of CD4 count alone (see Figure S5) were broadly similar with CD4 count graph of combining three metrics (see Figure S6). However, there is limited information in the Figure S6, with four different trajectories essentially showing a parallel upward trend, and the trajectory subgroups are consistent with baseline CD4 levels. While Figure S6 showed different trajectory groups had different CD4 recovery patterns, with fast speed of recovery in the group with high baseline CD4 level and slow speed of recovery in the group with low baseline CD4 level, which is consistent with conventional knowledge. Besides, Figure S6 shows the CD4 count and CD8 count changes and correlations behind different CD4/CD8 ratio recovery trajectories.

**Figure S5. CD4 count trajectories defined by the group-based trajectory analysis.**


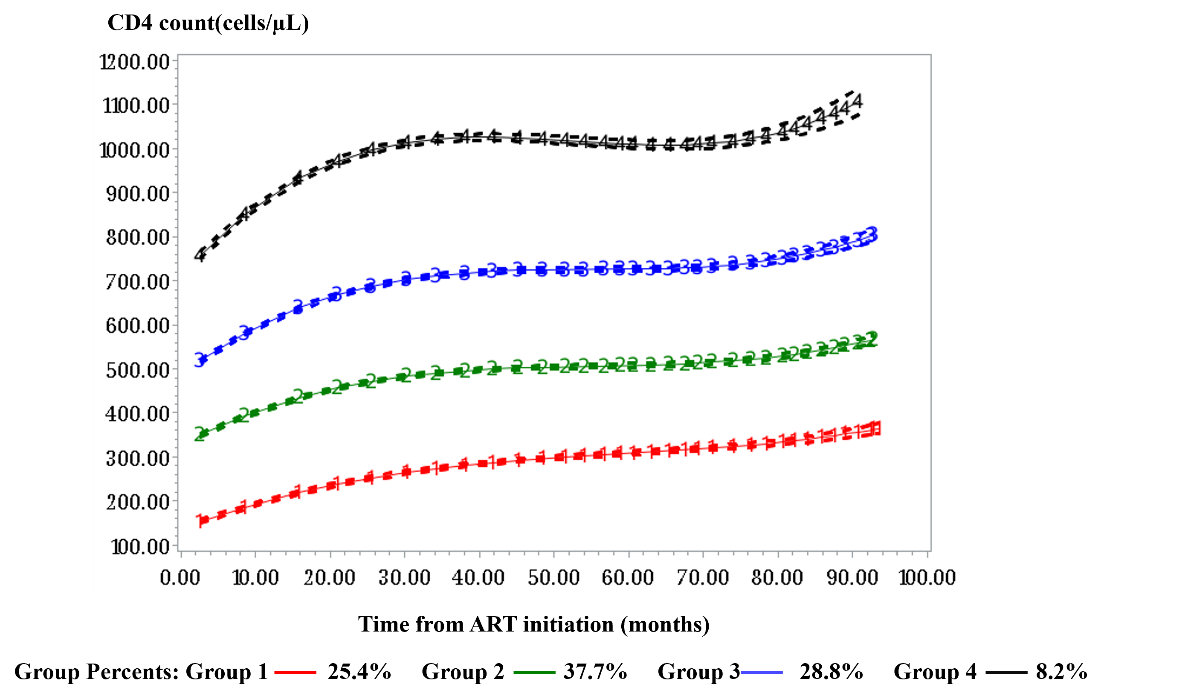


**Figure S6. CD4 count, CD8 count and CD4/CD8 ratio trajectories defined by the group-based multi-trajectory analysis.**


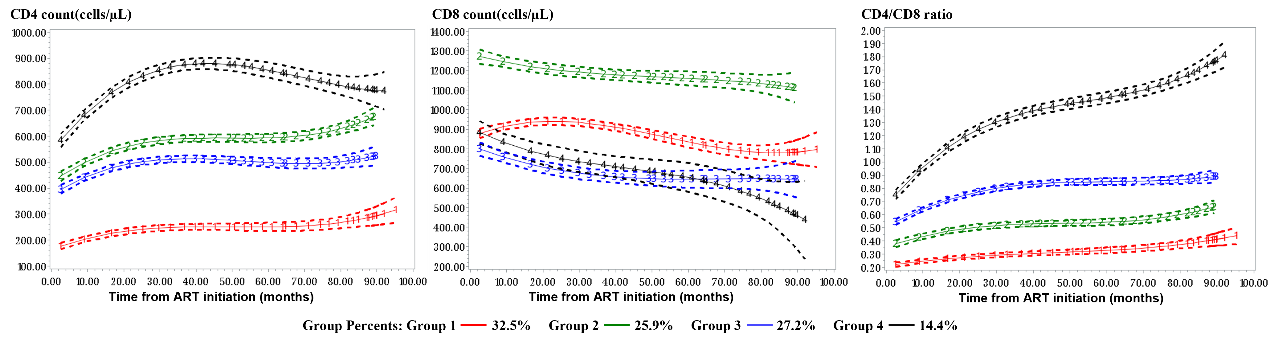


**4. Trajectory curves of CD4 count, CD8 count and CD4/CD8 ratio by means of observed data.**

As depicted in the figure below, the points represent the means of the observed data over time. This trajectory closely aligns with the curves estimated by the results of the group-based multi-trajectory model.


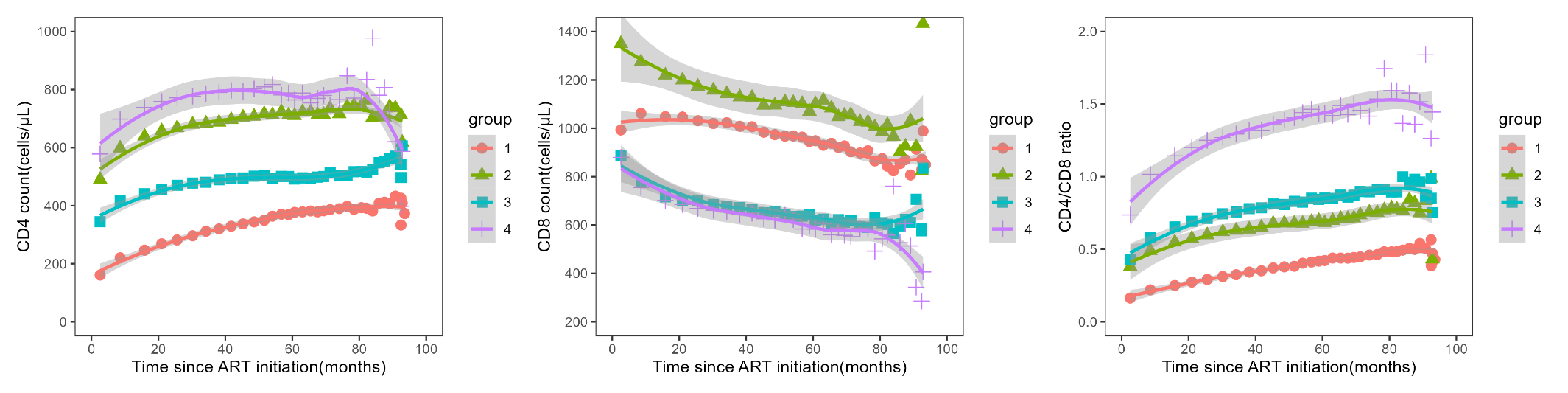


Figure S7. CD4 count, CD8 count and CD4/CD8 ratio trajectories defined by the means of observed data. CD4+ T-cell count (left column), CD8+ T-cell count (middle column), and CD4/CD8 T-cell ratio (right column) trajectories.

Abbreviations: ART, antiretroviral therapy.

The points and curves were drawn by the means of observed data.
